# Histidine Domain-Protein Tyrosine Phosphatase Interacts with Grb2 and GrpL

**DOI:** 10.1371/journal.pone.0014339

**Published:** 2010-12-15

**Authors:** Carmen-Alexandra Tanase

**Affiliations:** Department of Enzymology, Institute of Biochemistry, Bucharest, Romania; Emory Unviersity, United States of America

## Abstract

**Background:**

Histidine domain-protein tyrosine phosphatase (HD-PTP) plays a key role in vesicle trafficking and biogenesis. Although it is a large protein with at least five distinct structural domains, only a few of its interactors are presently known, and the significance of these interactions is largely obscure.

**Methodology and Results:**

In this study we performed a yeast two-hybrid screening using a human colon cDNA library and found that Grb2 and GrpL are binding partners of HD-PTP. Co-immunoprecipitation, pull-down and immunocytochemistry experiments confirmed the interactions. We also discovered that the central proline-rich and histidine-rich domain of HD-PTP is responsible for these interactions.

**Significance:**

The interaction of HD-PTP with two adapters of the Grb2 family, essential for numerous signaling pathways, suggests that HD-PTP might be important for signaling through a plethora of receptors.

## Introduction

Histidine Domain-Protein Tyrosine Phosphatase (HD-PTP), also known as non-receptor protein tyrosine phosphatase PTPN23, is a multidomain cytosolic member of the Bro1-domain-containing protein family. Besides its N-terminal Bro1 domain, HD-PTP has five other main structural domains: a V-domain with coiled-coil motifs, immediately after the Bro1 domain, a central unique proline-rich domain with numerous dispersed His residues (HD), a PTP-like domain (PTPc) and a second proline-rich domain towards the C-terminal end. Both the central and the C-terminal proline-rich domains have PEST motifs and appear to have disordered secondary structures. The PTPc domain was found to be catalytically inactive [1].

The multidomain structure of HD-PTP suggests that this protein might function as a multiadapter molecule. Recent data have shown the importance of HD-PTP to biogenesis of multivesicular bodies, vesicular trafficking [2], EGFR signaling [3], and focal adhesion turn-over [4], although the molecular mechanisms by which it affects these processes are still uncovered. In order to gain more insight on the functions of HD-PTP we sought to identify proteins with which it interacts. As a first step, we used a yeast two-hybrid system to screen a human colon cDNA library with the full length HD-PTP as bait. In this paper we report the identification of specific interactions of HD-PTP with two members of the Grb2 family adapters.

## Materials and Methods

### Cell culture and immunological reagents

Human cervical carcinoma HeLa cells were maintained in RPMI1640 medium (EuroClone) supplemented with 10% fetal bovine serum (FBS) and 2 mM L-glutamine. Human embryonic kidney cells HEK293T were maintained in Dulbecco's modified Eagle's medium (DMEM) supplemented with 10% FBS and 2 mM L-glutamine. All cells were cultured at 37°C in 5% CO_2_ humidified atmosphere. The following antibodies were used: rabbit anti-GFP (Abcam), goat anti-HA (Santa Cruz), mouse anti-GST (Sigma), mouse anti-Myc (Invitrogen), peroxidase-conjugated goat anti-rabbit and goat anti-mouse (GE Healthcare), peroxidase-conjugated donkey anti-goat (Santa Cruz), AlexaFluor 594-conjugated goat anti-mouse or rabbit anti-goat (Invitrogen).

### Constructs

For the preparation of the bait construct, the coding sequence of the full length human HD-PTP was subcloned into pBridgeLexA/v-src vector (a kind gift from Dr. Masaharu Noda, National Institute for Basic Biology, Okazaki, Japan), containing a LexA DNA binding domain. The subcloning strategy involved several steps. Briefly, using the vector pMObsFlag-HD-PTP [5](a generous gift from Dr. Mamoru Ouchida, University of Japan), we amplified by PCR two fragments of the coding sequence of HD-PTP: a first fragment of 535 bp contains the 5′-end of the coding region flanked by EcoRI and SalI restruction sites, and a second fragment of 798 bp contains the 3′-end flanked by NotI and SacII restriction sites. These fragments, along with the rest of the coding region of HD-PTP corresponding to the 3646 bp SalI-NotI fragment, were first subcloned in pBluescript SK+, to generate pBSSK(+) HD-PTP. The EcoRI-XhoI fragment containing the entire HD-PTP coding sequence from pBSSK (+)-HD-PTP was further inserted into pBridgeLexA vector digested with EcoRI and SalI. This construct contains the HD-PTP sequence in frame with LexA sequence according to the sequencing data. The sequences of the primers used for subcloning are listed in Table 1.

**Table 1:** PCR primer sequences.

Primer name	Sequence	Construct
EcoRI-HD-1	5′ aacgaattcccagccgccatggaggccgtgccccgc-3′	N-terminal full-length
SalI-HD-2	5′-tctggcggctcatgtcgacgct-3′	N-terminal full-length
NotI-HD-3	5′-tacctgcatcagcggccgctg-3′	C-terminal full-length
SacII-HD-4	5′-aaaccgcggcccctcgagtcaggtcttgttgagtgtcc-3′	C-terminal full-length
XhoI-Bro1-EGFP (For)	5′-aaactcgagcatggaggccgtgccccgcatgc-3′	Bro1-V, ΔHD
Bro1-STOP-EGFP	5′- aacgaattctcagcgggcagcctcgcgggcct-3′	Bro1-V
EcoRI-Bro1 (Rev)	5′- aacgaattcgcgggcagcctcgcgggcct-3′	ΔHD
EcoRI-PTPc (For)	5′-aacgaattccatggcggcactcagtctcct-3′	ΔHD
SacII-PTPc (Rev)	5′-aaaccgcggtcaggtcttgttgagtgtcc-3′	ΔHD
XhoI-HD (For)	5′-aaactcgagccagcagctcctggacagggagct-3′	HD
EcoRI-HD (Rev)	5′-aacgaattctcaatgctggctctccgggctgg-3′	HD, VHD
FOR-delta Bro1	5′-aaactcgagccagcagctcctggacagggagctgaa-3′	ΔBro1
REV-delta Bro1	5′-atgcgaattctcaggtcttgttgagtgtcc-3′	ΔBro1
Coiled-coil For	5′-aaactcgagaatgcagttggatcccgagacgg-3′	V-domain, VHD
Coiled-coil Rev	5′-aaagaattctcagcgggcctggcaggtgga-3′	V-domain
EcoRI-PTPc (For)	5′-aaagaattcctgcggcagttgcagcaggagc-3′	PTPc
SalI-PTPc (Rev)	5′-aaagtcgactcagctgatgcttgcactggcca-3′	PTPc
ΔPEST (For)	5′-gccaagctcagcattggccaaacagcgg-3′	ΔPEST
ΔPEST (Rev)	5′-ccgctgtttgccaatgctgagcttggc-3′	ΔPEST
BamHI-NcoI-GrpL (for)	5′-aaaggatccatggaagct-3′	pGAD-GrpL, pGEX-GrpL
XhoI-GrpL (rev)	5′-aaactcgagttatcgggtcatgggtgcca-3′	pGAD-GrpL, pGEX-GrpL

HD-PTP deletion variants were PCR amplified with *Pfu* DNA polymerase (Promega) using the same template pMObsr-Flag-HD-PTP. After PCR amplification, the fragments were restriction enzyme-digested and ligated into pEGFP-c2 vector (BD Biosciences) in frame with the EGFP sequence. The sequences of the primers are listed in Table 1. For making EGFP-ΔBro1 (705–1636) and EGFP-HD (705-1128), the fragments amplified using the primer sets FOR-delta Bro1/REV-delta Bro1 and XhoI-HD (For)/EcoRI-HD (Rev), respectively, were digested with EcoRI and XhoI and inserted between the corresponding sites of pEGFP-c2. The fragment 1–704 amplified with the primer set XhoI-Bro1_EGFP/Bro1-STOP_EGFP was digested with XhoI plus EcoRI and inserted between the corresponding sites of pEGFP-c2 to obtain EGFP-Bro1-V. The fragment 1171–1475, corresponding to the putative phosphatase catalytic domain was amplified with the primer set EcoRI-PTPc/SalI-PTPc, and inserted in pEGFP vector after digestion with EcoRI and SalI, to generate EGFP-PTPc construct. The EGFP-V-domain (401–700) and EGFP-VHD (401–1128) constructs were made by amplification with the primer sets Coiled-coil For/Coiled-coil Rev and Coiled-coil For/EcoRI-HD, respectively, further digestion with XhoI and EcoRI, and insertion in pEGFP-c2. For making the EGFP-ΔHD construct two fragments consisting of sequences 1–704 and 1221–1636 were amplified using the primer sets XhoI-Bro1-EGFP/EcoRI-Bro1 and EcoRI-PTPc/SacII-PTPc, respectively. The fragments digested with the XhoI plus EcoRI, or EcoRI plus SacII, respectively, were inserted between the corresponding sites of pEGFP-c2. This subcloning procedure introduced two amino acid residues (Glu-Phe) between the two HD-PTP fragments. The full length EGFP-HD-PTP was generated by subcloning from the pBS SK(+)HD-PTP described above by digestion with SacII and EcoRI and ligation into pEGFP-c2.

EGFP-ΔPEST vector was generated by site directed mutagenesis according to the QuickChange method (Stratagene) using the primers ΔPEST-For and ΔPEST-Rev and EGFP-HD-PTP as template. This deletion mutant lacks amino acid residues 1508–1595 of the human protein, region that includes two PEST motifs and several proline-rich motifs.

HA-Grb2 and GST-Grb2 were a generous gift from Dr. Jose M Rojas (National Center of Microbiology, Madrid, Spain). Myc-GrpL was a gift from Dr. Kevin E Draves (University of Washington, Seattle, USA). To generate GST-GrpL the full length GrpL cDNA was PCR amplified and subcloned in pGEX-6P-3 vector digested with EcoRI and XhoI restriction enzymes. To generate Activation Domain (AD)-GrpL, GrpL coding sequence was PCR amplified and subcloned in pGAD-DS vector digested with NcoI and XhoI. All constructs generated in this study were verified by DNA sequencing (MWG Biotech, Germany).

### Yeast two-hybrid screening

Strain: *Saccharomyces cerevisiae* L40: *MATa, trp1, leu2, his3, LYS2::lexA-HIS3, URA3::lexA-LacZ* (Invitrogen). Yeast two hybrid screening was performed using a human adult colon cDNA library in pGAD-DS, with selectable marker Leu and a GAL4 activation domain (Dualsystems Biotech). The bait construct pBridgeLexA-HD-PTP, containing the full length HD-PTP and Trp as selectable marker, was transformed into yeast strain L40 according to DUALhybrid kit user manual. After confirming the expression of the LexA-HD-PTP protein by Western blot with anti-LexA antibodies (Invitrogen) yeasts cells were further transformed with the prey vector pGAD-DS encoding a colon cDNA library according to Dualsystems protocol. The cells were plated on selective medium SD-Trp-Leu-His-Met to screen for interacting partners. His^+^ colonies appeared from 3 to 7 days after plating and were tested for β-galactosidase activity by the colony-lift filter assay using the substrate 5-bromo-4-chloro-3-indolyl-β-D-galactopyranoside. Only the colonies with a β-galactosidase activity developed within the first 5 h were selected. These positive colonies were re-streaked several times on selective media, and the individual isolated colonies were re-screened for β-galactosidase activity. These clones were also grown on SD-Trp-Leu-His in the presence of Met, which suppresses v-Src expression. The plasmids containing putative HD-PTP interaction partners were recovered from yeast and either transformed in *E.coli* or PCR amplified with primers specific for the pGAD-DS vector, namely, the commercially available GAD For and GAD Rev. The *E.coli* – amplified plasmids and the PCR products were sequenced. A BLAST search was carried out to identify the putative binding partners isolated in this screening.

### Recombinant protein production

*E.coli* BL21 cells were transformed with expression vectors containing the cDNAs for Grb2 and GrpL fused to the C-terminal end of Glutathione-S-Transferase (GST) and grown in Luria-Bertani medium with ampicillin. The expression of GST fusion proteins was induced with 0.3 mM isopropyl β-D-thiogalactopyranoside (IPTG) at A_600_ = 0.5 for 3 h at 37°C (for Grb2) or 6 h at 18°C (for GrpL). The expression of GST from pGEX-4T-1 vector was induced with 0.3 mM IPTG at A_600_ = 0.5 for 3 h at 37°C. Harvested bacterial pellets were sonicated in phosphate-buffered saline (PBS) containing 4 mM DTT and a cocktail of protease inhibitors (Roche). The extracts were cleared by centrifugation at 18,000xg for 30 min and incubated with glutathione-Sepharose 4B beads (BD Biosciences) for pull-down. The expression and the solubility of the recombinant proteins were determined by SDS-PAGE and Coomassie blue staining.

### GST pull-down and Western Blot

HEK293T cells in T-25 cell culture flasks were transfected with 5 µg DNA and 20 µl polyethyleneimine (Sigma) in complete medium. 20–24 h post-transfection, the cells were lysed in NP-40 lysis buffer (50 mM TrisHCl pH 8.0, 1.5 mM MgCl_2_, 10 mM NaF, 10 mM KCl, 150 mM NaCl, 1% NP-40) containing protease inhibitors for 30 min on ice. The lysates were cleared by centrifugation at 18,000xg for 30 min at 4°C. Cell lysates containing the full-length HD-PTP (100 µg total protein) were bound to GST, GST-GrpL or GST-Grb2 pre-adsorbed to glutathione-Sepharose beads for 1 h at 4°C. In the pull-down experiments with different constructs equal volumes of cell lysates containing 37.5-50 µg total protein were incubated with the same amount of GST-fusion protein bound to Sepharose resin. The protein complexes bound to GST-fusions were washed five times with NP-40 lysis buffer. Finally, the proteins were eluted in Laemmli sample buffer and separated by SDS-PAGE. The proteins were transferred to Immobilon P membrane (Millipore) using a semi-dry transfer system for 3–4 h at 60 mA. After blocking in 5% skim milk PBS with 0.05% Tween-20 (PBST), the membrane was incubated with rabbit polyclonal anti-GFP antibodies. Secondary antibodies were peroxidase-conjugated anti-rabbit IgGs. The blots were developed using SuperSignal West Femto Maximum Sensitivity Substrate (Thermo Scientific) and exposed on CL-XPosure Film (Pierce). The membranes were medium stripped with stripping buffer (1.5% Gly, 0.1% SDS, 1% Tween20, pH 2.2), PBS and Tris buffered saline containing 0.1% Tween 20 and incubated with anti-GST antibodies. Secondary antibodies were peroxidase-conjugated anti-mouse IgGs. The blots were again developed with SuperSignal West Femto Maximum Sensitivity substrate.

### GST Overlay assay

Cell lysates from HEK293T cells expressing different HD-PTP mutants (5.4–13.2 µg total protein) were fractionated by SDS-PAGE and blotted onto PVDF membranes. After blocking in 5% skim milk and PBST the membranes were incubated with 50 ng/ml GST-Grb2 or 100 ng/ml GST-GrpL in blocking solution overnight at 4°C. The next day the membranes were washed and incubated with anti-GST monoclonal antibodies for 1 h at room temperature. The blots were washed and incubated with horse radish peroxidase (HRP)-conjugated anti-mouse antibodies and finally developed by chemiluminescence with SuperSignal West Femto Maximum Sensitivity substrate. After medium stripping the membrane the blot was reprobed with anti-GFP antibodies. Following washes and binding of the HRP-labeled goat anti-rabbit antibodies the blots were developed by chemiluminescence.

### Immunoprecipitation

HEK293T cells were cotransfected with Myc-GrpL and either pEGFP-HD-PTP or pEGFP-c2 vector alone and lysed in NP-40 lysis buffer 24 h post-transfection. HEK293T cells cotransfected with HA-Grb2 and either pEGFP-HD-PTP or pEGFP-c2 were lysed in HEPES Lysis buffer (50 mM HEPES, pH 7.2, 150 mM NaCl, 1.5 mM MgCl_2_, 10% glycerol, 1% Triton X-100). Cleared lysates were incubated with anti-GFP antibodies and Protein A-Sepharose overnight at 4°C. After six washes with NP-40 lysis buffer, or HEPES lysis buffer, respectively, the proteins bound to resin were eluted in SDS-PAGE loading buffer. The samples were analyzed by SDS-PAGE and Western blotting with anti-Myc or anti-HA antibodies according to the protocol described above. The membrane was reprobed with anti-GFP antibodies after medium stripping.

### Immunofluorescence microscopy

HeLa cells were plated on glass coverslips on 6 well plates. After 12–24 h, the cells were transfected with the desired plasmids and Lipofectamine2000 (Invitrogen) in OptiMEM (Invitrogen). 18–24 h post-transfection the cells were rinsed briefly with PBS and fixed for 10 min in 4% paraformaldehyde at room temperature. The cells were permeabilized with PBS containing 0.2% Triton X-100 and further blocked with 1% BSA in PBS. The specimens were incubated for 1 h at room temperature with mouse anti-Myc or goat anti-HA antibodies in PBS containing 1% BSA. After washing three times in PBS, the coverslips were incubated with AlexaFluor 594-labeled goat anti-mouse or rabbit anti-goat antibodies for 30 min. Labeled cells were rinsed three times with PBS and mounted in *SlowFade* Gold antifade reagent with DAPI (Invitrogen). The cells were observed with a Nikon epifluorescence microscope and the pictures were obtained with NIS-Elements BR 3.0 software (Nikon).

## Results

### Identification of HD-PTP interacting proteins

**Figure 1:**
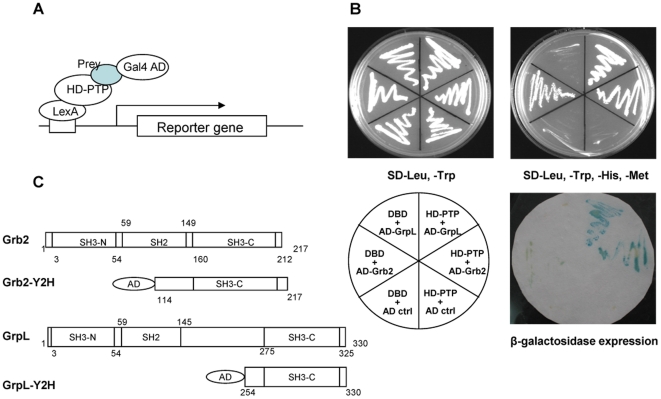
Identification of HD-PTP interaction with GrpL and Grb2 by yeast two-hybrid assay. (A) A scheme of the yeast two-hybrid screen. The binding of the “prey” to the HD-PTP “bait” yields a functional transcription factor that promotes the transcription of the reporter genes. In the absence of an interaction between the “bait” and the “prey” the reporters (e.g., *HIS3* and *LacZ*) are not induced. (B) Yeast L40 cells were co-transformed with the expression plasmid encoding the full-length HD-PTP fused with LexA DNA-binding domain (DBD, bait) and each expression plasmid encoding either the full length GrpL or the C-terminal half of Grb2 fused with the Gal4 activation domain (AD, prey). Transformants were selected on double dropout (SD-Trp, -Leu) medium plate. Each positive transformant was streaked on double dropout selection medium plate (SD-Trp-Leu) and quadruple dropout (SD-Trp,-Leu,-His,-Met). A filter replica of the SD-Trp,-Leu,-His,-Met plate was used to assay for β-galactosidase activity. Ctrl, empty vectors. (C) Schematic representation of the domain structure of Grb2, GrpL and the two interacting fragments found by the yeast two-hybrid screening.

To identify proteins that can associate with HD-PTP, we performed a yeast two-hybrid assay. The full length HD-PTP was cloned as a translational fusion with the LexA DNA binding domain and used as bait for screening a cDNA library fused with the GAL4 activation domain. A colon cDNA library was chosen because rat HD-PTP has been shown to be highly expressed in the digestive system [6]. The yeast two-hybrid screen was performed under conditions of v-Src kinase expression (minus Met in SD medium) when both the prey and the bait are potentially phosphorylated. We screened 7×10^5^ clones using SD-Trp-Leu-His-Met selection plates and found 74 positive clones that showed growth on selection plates and significant production of β-galactosidase. After selecting the unique clones by PCR amplification and restriction digestion we sequenced 20 independent clones. Interestingly, all the candidate interactors grew well both in medium with Met or without Met, suggesting that these interactions do not depend on Tyr phosphorylation. The sequence analysis and the homology search revealed that one of these positive clones corresponded to the C-terminal half (amino acid residues 114–217) of growth factor receptor-bound protein 2 (Grb2) and another to the C-terminal SH3 domain (amino acid residues 254–330) of Grb2-related protein of the lymphoid system (GrpL) (Fig. 1C). Grb2 and GrpL are members of the Grb2 family of adapter proteins that have a central SH2 domain flanked at the N- and C-terminal ends by SH3 domains. Fig. 1B shows the interaction of HD-PTP with the full-length GrpL and the C-terminal half of Grb2 in yeast two-hybrid assay. The pGAD-Grb2 vector contains also the 3′UTR sequence of Grb2. Although in the control assay the Grb2-containing vector was able to induce the HIS3 reporter, the fact that it did not induce the more stringent promoter LacZ argues that the interaction of Grb2 C-SH3 with HD-PTP is not a false positive. Moreover, the interaction of Grb2 with HD-PTP was confirmed in mammalian cells as discussed further.

### Association of HD-PTP with Grb2 and GrpL *in vitro* and in mammalian cells

**Figure 2:**
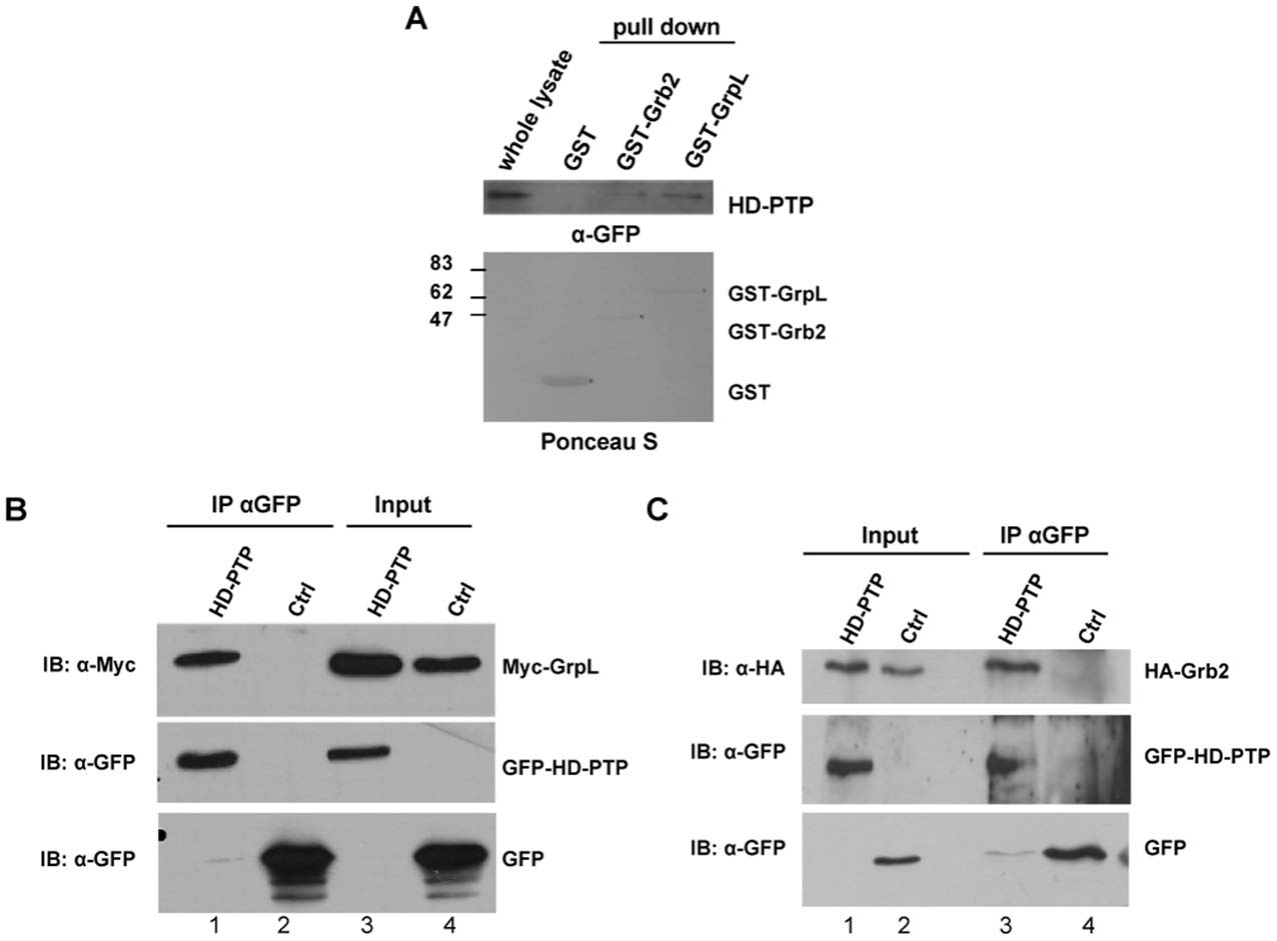
Analysis of HD-PTP interaction with Grb2 and GrpL by GST pull-down and immunoprecipitation. (A) HEK293T cells were transfected with EGFP-HD-PTP and cell lysates were incubated with GST, GST-Grb2, or GST-GrpL. After extensive washing, the presence of bound HD-PTP was analyzed by Western blot with anti-GFP antibodies. The input represents 20% of the lysate used in the pull-down assay. GST fusion proteins were visualized by Ponceau S staining. (B and C) HEK293T cells cotransfected with EGFP-HD-PTP or EGFP-c2 vector alone and either Myc-GrpL (B) or HA-Grb2 (C) were lysed and subjected to co-immunoprecipitation with anti-GFP antibodies. The co-immunoprecipitated proteins and aliquots of whole cell lysates were analyzed by Western blot with anti-Myc or anti-HA antibodies, respectively. The input represents 14% of the lysates used in co-immunoprecipitation.

To verify the yeast two-hybrid data we performed an *in vitro* GST pull-down assay. HEK293T cells expressing EGFP-HD-PTP fusion were lysed and the proteins were pulled down with purified GST-fusion proteins. We demonstrated that HD-PTP was not pulled-down with GST alone, but rather with GST-GrpL and GST-Grb2 (Fig. 2A). We also performed immunoprecipitation assays in mammalian cells. HEK293T cells were cotransfected with expression vectors containing EGFP-HD-PTP and either HA-Grb2 or Myc-GrpL. Myc-GrpL (Fig. 2B) and HA-Grb2 (Fig. 2C) coprecipitated with EGFP-HD-PTP. No interaction was detected when the empty vector pEGFP-c2 was cotransfected with either HA-Grb2 or Myc-GrpL. The faint EGFP band in the HD-PTP immunoprecipitation (lane 3 of Fig. 2C) was probably due to the partial degradation of EGFP-HD-PTP during protein extraction. The results demonstrated that Grb2 and GrpL interact specifically with HD-PTP in cultured mammalian cells.

**Figure 3:**
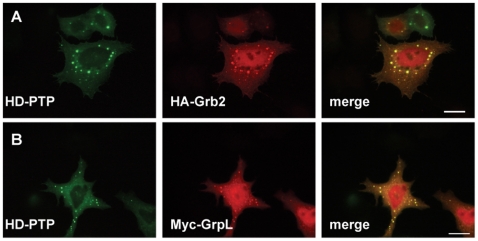
EGFP-HD-PTP partially co-localizes with HA-Grb2 and Myc-GrpL to endosomes. HeLa cells were transfected with plasmids expressing EGFP-HD-PTP and either HA-Grb2 (A) or Myc-GrpL (B). 18–24 h post-transfection, the cells were fixed, permeabilized and incubated with anti-Myc or anti-HA antibodies, followed by incubation with secondary antibodies labeled with AlexaFluor 594. Mounted coverslips were imaged with a fluorescence microscope. Scale bars, 20 µm.

Further, we examined the intracellular localization of HD-PTP and the two Grb2 family members. The expression plasmids for Myc-GrpL or HA-Grb2 were co-transfected with EGFP-HD-PTP plasmid into HeLa cells. As shown in Fig. 3 when co-expressed with HD-PTP, a population of Grb2 and GrpL colocalized with HD-PTP to large endosomes, while the rest was dispersed throughout the cytosol and in the nucleus. Our results do not exclude an interaction of Grb2 or GrpL and HD-PTP in the cytosol and at the plasma membrane, although we were not able to detect it due to technical limitations.

### The central proline-rich domain of HD-PTP binds to Grb2 and GrpL

**Figure 4:**
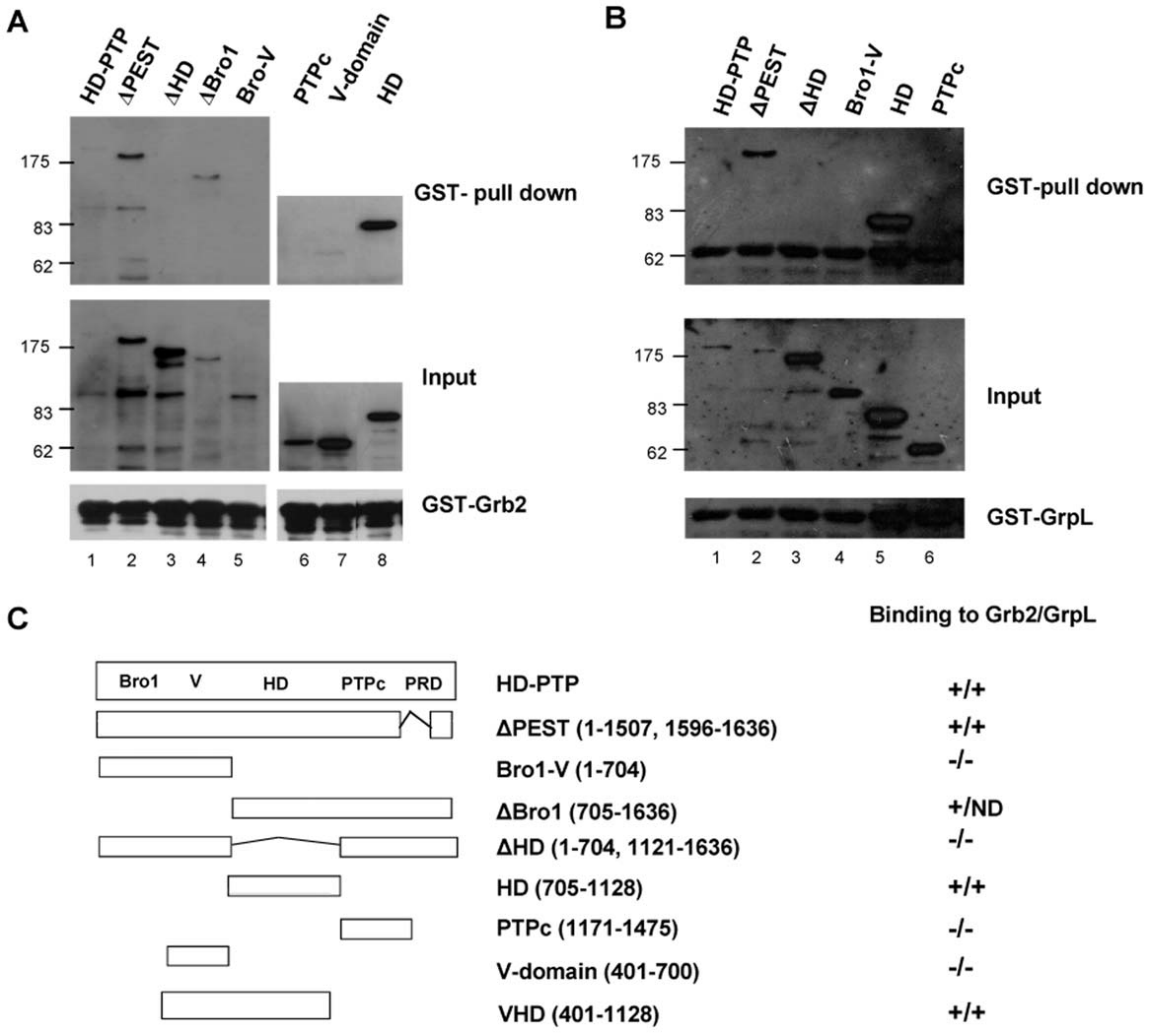
HD-PTP binds Grb2 and GrpL via its Histidine Domain. Lysates from HEK293T cells transfected with EGFP-HD-PTP were pulled-down with either GST-Grb2 (A), or GST-GrpL (B). Immunoblots for the pulled-down fractions, probed with anti-GFP antibodies are shown, along with the total lysates to demonstrate protein expression. Input represents 20% of each lysate used for pull-down. The figure shown is representative for experiments repeated at least twice with similar results. (C) Schematic overview of the HD-PTP constructs and the summary of the GST pull-down and GST overlay assays. PRD-proline-rich domain; ND-not determined.

**Figure 5:**
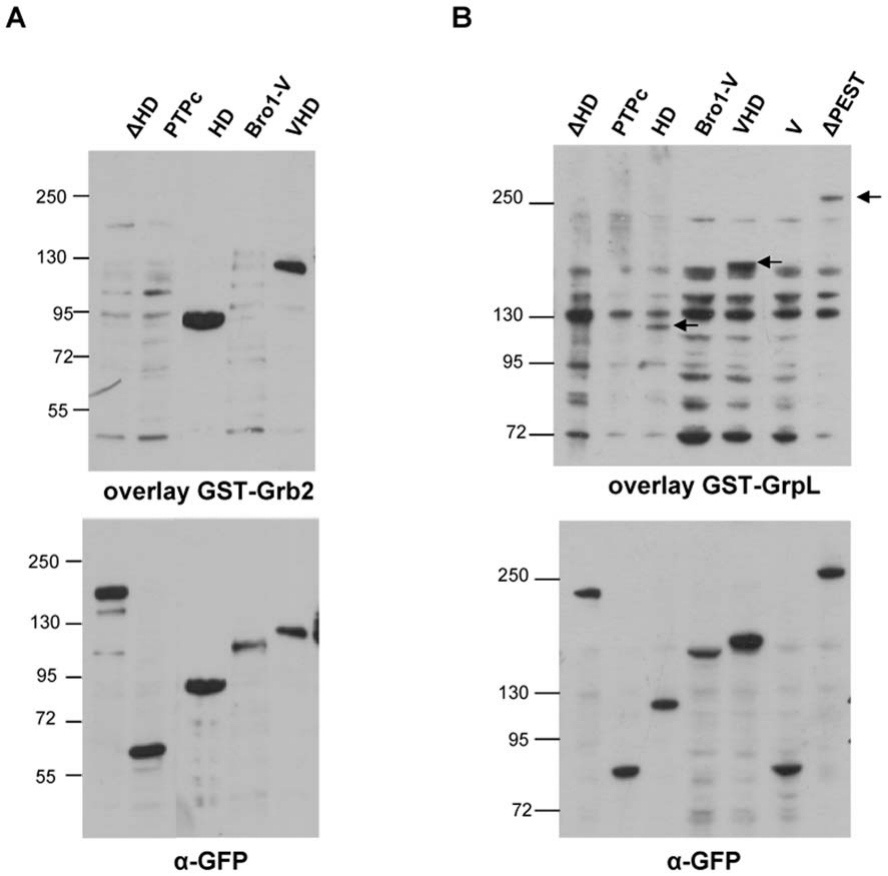
GST-tagged Grb2 and GrpL bind directly to mutants of HD-PTP that contain HD. Cell lysates from cells expressing different HD-PTP mutants were immobilized on PVDF membrane after SDS-PAGE. Overlays were carried out with 50 ng/ml GST-Grb2 (A) or 100 ng/ml GST-GrpL (B). PVDF membranes were probed with anti-GST antibodies (upper panels) and anti-GFP antibodies (lower panels). The specific bands in the GST-GrpL overlay are marked with arrows.

To identify which of the putative structural domains of HD-PTP interact with Grb2 and GrpL, *in vitro* pull-down assays were performed using affinity purified GST-Grb2 or GST-GrpL and lysates from cells transfected with EGFP-fusions of HD-PTP deletion mutants. As suggested by the presence of the proline-rich motifs, known to bind to SH3 domains, and by the interaction obtained in yeast two-hybrid of HD-PTP with the C-terminal SH3 domain of GrpL, we hypothesized that a region containing proline-rich motifs would be responsible for binding to the Grb2 family adapters. We observed that the Histidine Domain alone was pulled-down with the two adapters, whereas the construct lacking this domain was not (Fig. 4A and B). Moreover, other mutants containing the HD (i.e., ΔBro1, VHD, and ΔPEST) were also able to interact with the two Grb2-family members. We can conclude that the Histidine Domain (705–1128) was required and sufficient to bind to both Grb2 and GrpL. The observation that the deletion of the C-terminal proline-rich domain (in the ΔPEST construct) did not affect the binding of HD-PTP to Grb2 and GrpL, suggested that this domain is not required for HD-PTP binding to Grb2 family adapters. As expected, the Bro1, V- and PTPc domains, that do not contain proline-rich motifs, did not bind to the two Grb2 family members. Similar results were obtained by Far-Western analysis with GST-Grb2 and GST-GrpL (Fig. 5). We found that HD and VHD constructs bound the GST-Grb2, whereas ΔHD, PTPc and Bro1-V did not (Fig. 5A). Similarly, HD, VHD and ΔPEST constructs bound GST-GrpL whereas ΔHD, PTPc, Bro1-V and V constructs did not (Fig. 5B). Although the overlay assay with GST-GrpL showed a relatively high background we could still clearly observe that the deletion of HD resulted in a loss of binding to GrpL. The Far-Western results argue for a direct interaction between GrpL or Grb2 and the Histidine Domain.

**Figure 6:**
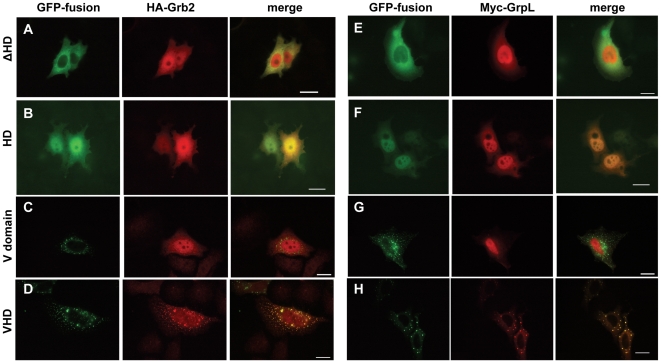
Histidine Domain is required for the co-localization of HD-PTP with HA-Grb2 or Myc-GrpL. HeLa cells were transfected with plasmids expressing EGFP-tagged HD-PTP proteins [ΔHD (A and E), HD (B and F), V-domain (C and G), or VHD (D and H)] and either HA-Grb2 (A–D) or Myc-GrpL (E-H). 18–24 h post-transfection, the cells were fixed, permeabilized and incubated with anti-Myc or anti-HA antibodies, followed by incubation with secondary antibodies labeled with AlexaFluor 594. Mounted coverslips were imaged with a fluorescence microscope. Scale bars, 20 µm.

The immunofluorescence analysis of HA-Grb2 or Myc-GrpL coexpressed with HD-PTP deletion mutants confirmed that the HD is essential for the interaction with the Grb2-family adapters. Thus, we found that the Grb2 and GrpL were dispersed throughout the cytosol and the nucleus when coexpressed with a HD-PTP mutant lacking its Histidine Domain (Fig. 6A and E). Grb2 and GrpL seem to colocalize with the Histidine Domain being dispersed throughout the cytosol and the nucleus (Fig. 6B and F). The expression of the V-domain alone, previously found to be necessary and sufficient for the endosomal targeting of HD-PTP [7], did not influence the intracellular localization of Grb2 and GrpL which remained dispersed throughout the cytosol and the nucleus (Fig. 6 C and G). In contrast, the expression of the VHD construct, that contains both the Grb2-interacting domain (HD) and the endosomal targeting domain (V-domain), led to the relocation of most of the cytosolic Grb2 and GrpL to endosomes labeled by the presence of EGFP-VHD (Fig. 6D and H).

## Discussion

HD-PTP, a Bro1 domain-containing protein, essential for early embryo development [8], has very poorly understood functions. Being a large protein with 1636 amino acids and several putative structural domains it is likely that it can interact with numerous functional partners. However, only few partners have been identified so far. The center proline-rich Histidine Domain binds to endophilin A1, an SH3 protein involved in receptor endocytosis and signal transduction and to Tsg101, a component of Endosomal Sorting Complex Required for Transport (ESCRT)-I [9]. The same Histidine Domain binds in a Ca^2+^-dependent manner, to ALG-2, a protein important for apoptosis [9]. The Bro1 domain interacts with CHMP4b, a component of ESCRT III [2], [9]. Furthermore, HD-PTP appears to interact *in vivo* with the focal adhesion kinase and to regulate both the kinase's tyrosine phosphorylation state and its intracellular localization [10]. These interactions indicate that HD-PTP might play roles in endosomal protein sorting and trafficking, apoptosis, and cell adhesion.

In this study, we describe two novel interactions of HD-PTP. Using a yeast two-hybrid approach, we found that Grb2 and GrpL, two members of the Grb2 family adapters, are binding partners of HD-PTP. The Grb2 family consists of a central SH2 domain flanked by two SH3 domains. Through its SH2 domain, which is a conserved sequence of 90 amino acids, Grb2 can interact directly with receptor tyrosine kinases (RTKs) (e.b., epidermal growth factor receptor, hepatocyte growth factor receptor, etc.) and non-receptor tyrosine kinase, such as focal adhesion kinase (FAK), as well as substrates of tyrosine kinases, via preferential binding to the phosphopeptide motif pYXNX (where N is asparagines and X any residue) [11]. The C- and N-terminal SH3 domains, which have a conserved sequence of around 50 amino acids, bind proline-rich regions within the interacting proteins. In addition to these three domains, GrpL has a proline/glutamine rich region of 135 amino acids between the SH2 domain and the C-terminal SH3 domain. The sequence analysis of the isolated preys reported here revealed a fragment that contains the C-terminal half of Grb2 consisting of the entire C-SH3 domain and a short fragment of the SH2 domain, and the C-terminal end of GrpL containing the entire C-SH3 domain and a short peptide of the linker between the SH2 and C-SH3 domains. These data suggest that the C-terminal SH3 domains of both GrpL and Grb2 are sufficient for their interaction with HD-PTP in yeast two-hybrid assay. Moreover, this interaction seems to not require the phosphorylation by v-Src, since the colonies selected in conditions of v-Src expression (SD-Trp,-Leu,-His,-Met) were able to grow also under conditions in which v-Src expression was repressed (i.e., on SD-Trp,-Leu,-His). The interactions between Grb2 or GrpL and HD-PTP were verified in mammalian cells using coimmunoprecipitation, *in vitro* pull-down and immunofluorescence assays. We found that Grb2 and GrpL were able to bind *in vivo* and *in vitro* to the full length HD-PTP, and that Grb2 and GrpL partially colocalized with HD-PTP to cytosolic vesicles. While this manuscript was in preparation Harkiolaki et al., 2009 [12] reported that HD-PTP could be pulled down with the C-terminal SH3 domain of GrpL. This result supports our findings. Since HD-PTP is a substrate for Src kinase [13] and Grb2 and GrpL contain SH2 domains that bind peptide motifs containing phosphorylated tyrosines, it is conceivable that phosphorylated HD-PTP might interact also with the SH2 domains of these adapters. Further experiments are required to address this hypothesis and to determine whether HD-PTP could also bind to the N-terminal SH3 domain of the adapters.

Here we also show that the Histidine Domain (amino acid residues 705–1128) is indispensable for the interaction with Grb2 and GrpL *in vitro*. Thus, by GST pull-down and Far-Western experiments we found that GST-Grb2 and GST-GrpL were able to bind to the Histidine Domain alone, but not to a construct lacking this domain. At the same time the results show that the C-terminal proline-rich domain is not involved in binding the Grb2 family adapters. The deletion of this domain (in EGFP-ΔPEST) did not prevent the interaction seen with the full length protein and its presence in the context of a protein lacking the HD (i.e., the ΔHD construct) did not result in an interaction with the adapters. Our results also show that three other domains of HD-PTP, namely the N-terminal Bro1 domain, the V-domain and the PTPc domain do not interact with Grb2 or GrpL under the conditions used in this study. However, it is plausible that any of these domains, which contain putative tyrosine phosphorylation sites, might interact with the SH2 domains of Grb2 adapters when phosphorylated. In summary, our data demonstrate that HD-PTP interacts with Grb2 and GrpL through the SH3 domain-binding motifs present in the Histidine Domain. The immunofluorescence data support this conclusion showing that when coexpressed with a mutant that contains only the Histidine Domain and the V-domain, GrpL and Grb2 relocate to cytoplasmic vesicles where they colocalize with the HD-PTP mutant. In contrast, when coexpressed with deletion mutants lacking the Histidine Domain, the two Grb2 adapters have a dispersed cytosolic and nuclear localization. The overexpressed Histidine Domain appeared diffusely located throughout the cytosol and the nucleus, similar with the two adapters. These results are supported by Harkiolaki et al., 2009 [12] where the interaction motif of HD-PTP with the C-SH3 domain of GrpL has been mapped to amino acids 717–725 (PPRPTAPKP) within the putative Histidine Domain. The Histidine Domain of HD-PTP has a unique sequence that contains 16 His and 2 Cys residues linked by proline-rich elements of 3 to 51 amino acid residues each. According to ELM prediction the Histidine Domain has 35 putative SH3 domain-binding motifs [14]. It remains to investigate which of these motifs bind to Grb2. Within this domain there are binding motifs for Tsg101 and endophilin A1, two proteins involved in endocytosis [9]. The putative Tsg101 binding motif, _720_ PTAP _723_ (motif also found in Alix, another member of the Bro1 domain-containing proteins that has a domain structure similar to the N-terminal half of HD-PTP), overlaps with the GrpL binding motif. This suggests that the binding of Tsg101 and GrpL to HD-PTP are mutually exclusive.

Grb2 is a ubiquitously expressed adapter that acts as a critical downstream intermediary in several signaling pathways, including growth factor receptors pathways [15]–[18]. In receptor tyrosine kinase (RTK)/Ras pathway Grb2 adapter mediates the activation of Ras by bringing the guanine nucleotide exchange factor Sos1 to the plasma membrane adjacent to the membrane-associated Ras. There activated Ras alters the activity of several effectors, such as Raf kinase, phosphatidylinositol-3 kinase, and Ral GTPase [19]. Following the rapid and transient signaling initiated at the plasma membrane, RTKs are internalized and targeted to endosomal compartments where they can continue signaling [3], [20]. Grb2 and Ras accompany these receptors to the endosomes [21], [22]. At the endosomal membrane the activated RTKs are sorted and internalized in intralumenal vesicles, processes that end their signaling activity and generate multivesicular bodies. The internalized receptors are transported to the lysosomes for degradation. Cytosolic complexes such as ESCRTs and their associated proteins are involved in these highly dynamic and regulated processes. Grb2 seems to be involved in these processes as well, since mutations in either of its SH3 domains impede the epidermal growth factor receptor trafficking from the early to the late endosomes and the formation of the multivesicular bodies [23]. Interestingly, a similar phenotype was observed when HD-PTP levels were knockdown in cell culture [2]. The mechanisms by which Grb2 regulates endocytosis of RTKs is not fully understood, and we can hypothesize that HD-PTP and Grb2 work together, probably with other proteins as well, to assemble and/or coordinate the assembly of endosome-associated protein complexes essential for vesicle biogenesis and protein sorting.

GrpL, also known as Gads, Grap2, Mona and Grf40, is expressed only in hematopoietic tissues, including bone marrow, lymph node, and spleen [24]. Both Grb2-family adapters found to bind to HD-PTP are important regulators in lymphocytes signaling and development [24]–[29]. T-cell receptor (TCR) engagement with anti-CD3 antibodies or peptide MHC complexes induces a cascade of Tyr phosphorylations, which leads to the fast recruitment and subsequent activation of downstream effectors of the TCR/CD3 activated complex. Adapter proteins such as LAT become phosphorylated on multiple Tyr residues. Phosphorylation of LAT creates binding sites for SH2 domains of other proteins, including phospholipase C γ1 (PLC-γ1), Grb2, GrpL and Grap [30]–[32]. Thus, SLP76, which is constitutively bound to GrpL is brought to the TCR signaling complex at the plasma membrane [30]. In addition, Grb2 recruits Sos1 and E3 ubiquitin ligase c-Cbl, which are bound to its SH3 domains [33]. These interactions are crucial for the regulation of calcium signaling in T cells [28], [34] and for coupling the TCR to Ras through a pathway involving PLC-γ1, Tec family kinases, and RasGRP [35]. c-Cbl mediates the ubiquitination of TCRζ chain leading to TCR internalization into endosomal compartments and subsequent degradation of the receptor in activated T cells [36]. c-Cbl also mediates the segregation of LAT/GrpL/SLP-76- containing microclusters from activated TCR/CD3 complexes and further induces their endocytosis [37]. It is conceivable that these endocytozed microclusters contain other adapters and enzymes associated with activated LAT. Our results suggest that HD-PTP may be one of the adapters associated with LAT upon TCR activation and that it may modulate the endocytic trafficking of LAT/SLP-76 microclusters, thus downregulating the signaling output of the TCR. Further experiments are required to elucidate the molecular mechanisms controlled by HD-PTP in lymphocytes.

In conclusion, we have identified Grb2 and GrpL as binding partners of HD-PTP. These interactions with adapters, which are essential for numerous signaling pathways, suggest that HD-PTP might have a role in the regulation of downstream events of a plethora of receptors.
